# Disease of the Lungs

**Published:** 1902-03-01

**Authors:** 


### DISEASE OF THE LUNGS.

TUBERCULOSIS—(*Continued from page* 360).

**Treatment.**—A preliminary note on the so-called "silver treatment" of phthisis by intravenous injections of protargol comes from Ewart.[14] He "was led to substitute protargol for formaldehyde injections by the conspicuous success of the administration of nitrate of silver in pneumonia (Caccianiga) and of its subcutaneous injection in phthisis (Mays)." Ewart finds that its action is more decided, more rapid, and more lasting than formaldehyde. The clinical effects are "a remarkable subjective feeling of improvement coinciding with manifest improvement in aspect and in strength, a rapid diminution in the cough and in the expectoration, and a more or less rapid change from the dense sputum of phthisis to simple catarrhal sputum and ultimately to hyaline mucus, etc." The injection consists of 40 cc. of saline solution containing $1\frac{1}{2}$ to $2\frac{1}{2}$ grains of protargol, and this is preceded and followed by an injection (through the same needle) of a few cc. of pure saline solution to obviate leakage of irritating fluid into the tissues. From 12 to 15 injections generally suffice, best administered at intervals of one day. The method is only in its infancy; let us trust the hopes of the author may in some measure be realised. Buck[15] reports a case of extensive lupus of the lip and in the nose treated with urea, according to the suggestion of Harper for tuberculosis. Twenty grains, three times a day, was given at first, gradually increased to 30 and 40 and even to 60 grains thrice daily. Healing of the ulcers soon commenced, and advanced almost to completion, and the discharge from the nasal cavity ceased.

**Pneumomycosis.**—In recording one of these rare cases, Buttar[16] states of the condition of the patient, a female aged 52 years, that she was much troubled by cough, wheezing, and shortness of breath. Her temperature was 99° Fahr.; her respiration was rather laboured, the distress becoming much plainer on any exertion. She could not lie on her back, because of the difficulty of breathing. She said she was much worse at night. The lips were slightly blue. Her finger-tips were also blue, but not clubbed. The tongue was coated with a thick yellowish-white fur. There was very little in the lungs except a few moist sounds at the bases. From records of other cases and his own experience of this case he concludes:—(1) That the oidium albicans, like the aspergillus fumigatus, may develop in the lungs. (2) That the organism apparently develops in a previously unhealthy lung, or after some disease lowering the vitality of the patient. (3) That the resulting complication leads to a tedious illness of one or two months' duration; but at the same time the disease is much less serious than the pneumomycosis due to the aspergillus, inasmuch as it leaves no fresh lesion of the lung. (4) That the symptoms are chiefly those of bronchitis. (5) That the diagnosis can only be made by an examination of the sputum.

**Empyema.**—One of the great defects of simple incision of the costal pleura and chest walls, followed by drainage of empyemic cavities, is the persistence of lung retraction, the failure of the lung to expand being due to the concomitant thickening of the pulmonary pleura. To overcome this drawback Delorme incises the pulmonary pleura. A case illustrating the good results which follow this device is recorded by C. L. Dowd.[17] After resection of a portion of the seventh rib, efforts were made to expand the lung for three and a half months. As the child was not doing well after this long treatment, a secondary operation was done. About three inches of the ninth rib, and smaller portions of the ribs above this as far as the third, together with a corresponding wedge-shaped piece of costal pleura, were removed. This gave a very good exposure of the thickened pulmonary pleura, through which an incision about four inches long was made, and the pleura pushed back from each side. The lung was then seen to expand to a considerable degree, but not as satisfactorily as Delorme describes in speaking of his cases. He states that after his incision the lung expands as an animal's lung does when it is inflated. Drainage was carried from the anterior opening between the second and third ribs to this large lateral opening. This was the third case in which Dowd had incised the pulmonary pleura. One of the cases was complicated with miliary tuberculosis and the lung did not expand. In that case the pleura could not be peeled from the lung tissue and the operation was without benefit. In the second case the pleura was very thin and the lung expanded satisfactorily. The case promised very well at first and the wound healed, but opened again at a later time, and the patient has since developed tuberculosis in other regions of the body. Dowd's operation differed somewhat from the Delorme method in the removal of a portion of the chest wall instead of lifting it as a flap and then returning it to its position.

[14] Merck's Monthly, Sept. 28, 1901. [15] Practitioner, 1901, No. lxvii. [16] West Lond. Med. Jour., July 1901. [17] Annals of Surg., July 1901.

